# Arterial Spin Labeling MRI for Predicting Microvascular Invasion of T1 Staging Renal Clear Cell Carcinoma Preoperatively

**DOI:** 10.3389/fonc.2021.644975

**Published:** 2021-05-18

**Authors:** Han-Mei Zhang, Da-Guang Wen, Yi Wang, Yi-Ge Bao, Yuan Yuan, Yun-Tian Chen, Bin Song

**Affiliations:** ^1^ Department of Radiology, West China Hospital, Sichuan University, Chengdu, China; ^2^ Department of Urology, West China Hospital, Sichuan University, Chengdu, China

**Keywords:** arterial spin labeling MRI, renal blood flow, microvascular invasion, diagnostic test (MeSH), renal clear cell carcinoma

## Abstract

**Background:**

Microvascular invasion (MVI) is a valuable factor for T1 staging renal clear cell carcinoma (ccRCC) operation strategy decision, which is confirmed histopathologically post-operation. This study aimed to prospectively evaluate the performance of arterial spin labeling (ASL) MRI for predicting MVI of T1 staging ccRCC preoperatively.

**Methods:**

16 volunteers and 39 consecutive patients were enrolled. MRI examinations consisted of ASL (three post label delays separately) of the kidney, followed by T1 and T2-weighted imaging. Two sessions of ASL were used to evaluate the reproducibility on volunteers. Renal blood flow of renal cortex, medulla, the entire and solid part of the tumor were measured on ASL images. Conventional imaging features were extracted. MVI and WHO/ISUP classification were evaluated histopathologically. A paired t‐test was used to compare the renal cortex and medulla between ASL 1 and ASL 2. The reproducibility was assessed using the intraclass correlation. Differences in mean perfusion between the entire and the solid parts of tumors with or without MVI were assessed separately using Student’s t test. The diagnostic performance was assessed. Logistic regression analysis was used to indicate the independent prediction index for MVI.

**Results:**

The two sessions of ASL showed no significant difference between the mean cortex values of RBF. The cortical RBF measurements demonstrated good agreement. 12 ccRCCs presented with MVI histopathologically. Mean perfusion of the solid part of tumors with MVI were 536.4 ± 154.8 ml/min/100 g (PLD1), 2912.5 ± 939.3 ml/min/100 g (PLD2), 3280.3 ± 901.2 ml/min/100 g (PLD3). Mean perfusion of the solid part of tumors without MVI were 453.5 ± 87.2 ml/min/100 g (PLD1), 1043.6 ± 695.8 ml/min/100 g (PLD2), 1577.6 ± 1085.8 ml/min/100 g (PLD3). These two groups have significant difference at all the PLDs (p < 0.05). The RBF of PLD1 of the solid part of tumor perfusion showed well diagnostic performance for predicting MVI: sensitivity 75%, specificity 100%, positive predictive value 66.7%, and negative predictive value 95.7%. The maximum diameter of the tumor, ill-defined margin, and the solid part of tumor perfusion were the independent prediction index for MVI.

**Conclusion:**

ASL MR imaging has good reproducibility for renal cortex, and good diagnostic performance for predicting MVI for ccRCC.

## Introduction

With the imaging technology developing, the detection of renal cell carcinoma (RCC) has increased (1). RCC is the most common type of kidney solid tumor, accounting for 90% of malignant kidney tumors (2). For focal RCC T1/2N0M0, although cryoablation is an optional therapy, high quality evidence suggests that surgery is the best therapy strategy. The operation strategy mainly contains radical nephrectomy (RN) and partial nephrectomy (PN). Many retrospective studies (3–5) have shown that from the perspective of oncology and patient quality of life for RCC tumors less than 4 cm in diameter RCC, partial resection is more likely to be chosen. In the Kidney Cancer Management Guide, for RCC with a tumor diameter greater than 4 cm and less than 7 cm, there is no high-quality evidence to support which procedure (RN or PN) should be chosen.

It is also mentioned in the guidelines that for patients with venous tumor thrombus, radical nephrectomy is currently recommended (4). It has been suggested that RCC with a diameter greater than 4 cm has a larger proportion accompanied with renal vein thrombosis than RCC smaller than 4 cm (6). Sugino et al. (7) suggest that the first step in the invasion of tumor cells into large blood vessels is to grow in the efferent veins (*i.e.* venules) and then gradually spread to the large and medium veins. The venules belong to the category of microvascular, and the microvascular are blood vessels that can only be seen under the microscope. It should include tiny genus of large veins, tiny venous vessels in the tumor capsule, and tiny segments within the tumor fibers. This means that microvascular invasion is the former step of venous tumor thrombus. In this situation, radical nephrectomy is preferred. In particular, previous studies reported that MVI is an independent risk factor of tumor recurrence after surgical resection of hepatocellular carcinoma (8). Furthermore, multiple research studies (9–11) have shown that microvascular invasion is an important factor in the prognosis of renal cell carcinoma. Therefore, if microvascular invasion can be accurately predicted before surgery, the operation strategy for patients with renal cell carcinoma (especially the size larger than 4 cm and smaller than 7 cm) will be more confident; the management of renal cell carcinoma is a step closer; it can also provide more information for predicting the prognosis of patients before surgery.

Some conventional MRI features have been reported to indirectly predict MVI, such as irregular tumor margins, capsule disruption, peritumoral enhancement (12), lack of the functional MRI features, which may occur before conventional MRI features and supply more functional information. Arterial spin labeling (ASL) is a magnetic resonance imaging (MRI) technique that allows the non-invasive quantitative assessment of tissue perfusion, using the magnetization of endogenous-labeled blood to provide contrast, without using of any exogenous contrast agent (13). The basis for the calculation of tissue perfusion is the difference between images acquired with and without labeling. Tissue perfusion obtained with ASL MRI is not affected by vessel permeability and is beneficial for eliminating the risk of nephrogenic systemic fibrosis in patients with renal impairment (14, 15). ASL for renal blood flow quantification in healthy kidneys, renal allografts, and renal masses has good feasibility and reliability (16, 17).

Since microvascular invasion is tightly related with the invasiveness of tumor, which are usually reflected from the perfusion of tumor, this study aimed to prospectively evaluate the performance of functional ASL imaging for predicting microvascular invasion of T1 staging (maximum diameter ≤7cm) renal clear cell carcinoma which was confirmed histopathologically.

## Materials and Methods

### Inclusion and Exclusion Criteria

The hospital institutional review board permitted this study, and all patients have signed the written informed consent. 16 volunteers and 39 consecutive patients were enrolled between August 2019 and June 2020. The inclusion criteria were: (1) renal lesion (maximum diameter ≤7 cm) was detected by abdominal computed tomography (CT) or ultrasound; (2) no treatment of the renal lesion; (3) age between 18 and 70 years old; (4) good image quality; and (5) radical nephrectomy. The exclusion criteria of the patients were as follows: (1) contraindication to MRI; (2) post-surgical histopathology indicated no ccRCCs (18).

### MRI Protocol

MRI examinations consisted of ASL MRI of the kidney, followed by T1-weighted and T2-weighted imaging of the abdomen, as described in **Table 1**. Ten healthy volunteers were scanned using a breath-hold renal perfusion imaging using Pseudocontinuous ASL (pCASL) scheme on a 3 T GE W750 scanner. Spin-echo (SE)-based Echo planar imaging (EPI) is used for readout techniques. The time between the inversion pulse to the central k-space of the first slice was defined as post-label delay. Three different PLDs (500, 1,000, and 2,000 ms) were used separately. Seven slices covering both kidneys were acquired with an oblique-coronal image orientation. The total scanning time is 2 min 13 s.

**Table 1:** Imaging parameters for ASL-MRI, T1WI, T2WI sequences.

Parameter	ASL imaging	Coronal T2WI	Axial T2WI	Axial T1WI
Repetition time (ms)	5,323.8	1,000	1,650	150
Echo time (ms)	18	68	26	1.4/2
Flip angle (degrees)	/	150	111	75
Section thickness (mm)	7	3	5	5
Matrix	64 × 96	320 × 256	320 × 256	256 × 205
Field of view (mm)	320 × 320	452 × 452	378 × 276	438 × 285
Acquisition time	2 min 13 s	27 s	1 min 7 s	33 s
Post label delay (ms)	500 (PLD1), 1,000 (PLD2), 2,000 (PLD3)	/	/	/

A second identical scan session within 1 h after the first scan session (ASL 1 and ASL 2) for all volunteers (without eating or drinking) was conducted. Patients included in this study were scanned with the same protocol.

### Quantification of Renal Cortex Perfusion and Renal Mass

Analysis was performed using custom-written MATLAB programs (The MathWorks, Inc., Natick, MA, USA). ASL perfusion-weighted (PW) difference images were formed by subtracting the non-selective images from the selective images (19). PW difference images were inspected for motion, misaligned pairs discarded, and the remaining PW difference images averaged to form an average PW difference image (ΔM) for each slice. These were then normalized to the base M0 image. T1 maps were formed by fitting the inversion recovery data to a two-parameter model. ΔM, T1, and base M0 maps were used to generate a renal perfusion (f) map, in units of ml/100 g/min, by fitting the data to a kinetic model. Each slice was fitted taking into account the exact post-label delay at which the slice was collected following the labeling pulse.

Renal blood flow in the area of renal cortex, medulla, the entire and solid parts of the tumor were measured on ASL images using region-of-interest (ROI) analysis by one reviewer (18). Image J was used to draw ROI to measure the perfusion and show the pseudocolor map. The reviewer was blinded to the histopathological analysis. The ROI of tumor was drawn covering the entire and solid parts (avoiding the necrosis area) of tumor and did not exceed the contour of the tumor. The ROI of the renal cortex was drawn covering the cortex.

### Conventional Imaging Features of Renal Mass

Conventional imaging features were extracted including the size of the tumor (which was quantified in the maximum section in axial image), signal features on T1WI and T2WI, the smooth/ill-defined margins, the shape (spherical, elliptical, irregular), presence of necrosis (20, 21) (**Table 2**).

**Table 2:** Image features of kidney tumors.

Kidney Tumor Location		group1*	group2#	T1WI signal		group1	group2
left	20	7	13	low	20	7	13
right	19	5	14	medium	15	4	11
				high	4	1	3
T2WI signal		group1	group2	central necrosis		group1	group2
low	3	1	2	presence	17	5	12
medium	8	3	5	absence	22	7	15
high	28	8	20				
margin		group1	group2	the shape		group1	group2
smooth	17	8	9	spherical	15	5	10
ill-defined	22	4	18	elliptical	13	4	9
				irregular	11	3	8
the size of the tumor		group1	group2				
		3.6 ± 2.1	4.7 ± 1.8				

*group1: kidney tumors with microvascular invasion.

#group2: kidney tumors without microvascular invasion.

### Histology

According to the “Guideline of Standardized Pathological Diagnosis of Primary Liver Cancer (2015 edition)”, MVI was defined as “a cancer cell nest with >50 cells in the endothelial vascular lumen under microscopy”, which was classified into three grades according to the number and distribution of MVI: M0, no MVI; M1 (the low-risk group), ≤5 MVI in adjacent liver tissue ≤1 cm away from the tumor; M2 (the high-risk group), >5 MVI or MVI in liver tissue >1 cm away from the tumor (22). This study used the above standard to evaluate the MVI of ccRCC. WHO/ISUP classification was also evaluated.

### Statistical Analyses

SPSS 19.0 software (SPSS, Chicago, IL, USA) was used for statistical analysis. The ASL perfusion values obtained were used for quantitative analysis. The mean, standard error of the mean and 95% confidence interval (CI) of perfusion of renal cortex perfusion and renal tumors were calculated.

Perfusion of the renal cortex and medulla was compared by a paired t-test between ASL 1 and ASL 2. The intraclass correlation (ICC) was used to assess the reproducibility (23).

Differences in mean perfusion between the entire and the solid parts of the tumors with or without microvascular invasion were assessed separately using Student’s t-test. The receiver operating characteristic (ROC) curve analysis was conducted to assess the diagnostic power. The diagnostic performance of ASL imaging for predicting MVI of the ccRCC was assessed using the diagnostic test index: sensitivity, specificity, positive predictive value, and negative predictive value.

Logistic regression analysis included the entire and solid parts of tumor of perfusion, conventional MR imaging features, ISUP grading, and indicated which index was the independent prediction index for RCC accompanying with MVI.

## Results

Image acquisition was completed successfully in 16 volunteers for 32 kidneys (eight males and eight females, age range, 31–58, mean age, 44.3 ± 13.9 years) and 39 patients (22 males and 17 females, age range, 43–70, mean age, 54.3 ± 13.6 years) for 39 renal lesions. Histopathological diagnosis was obtained by radical nephrectomy. The mean interval between MRI and histological analysis was 2.1 ± 1.2 days. 39 patients with 39 lesions were diagnosed ccRCCs. 12 cases presented with MVI and the other 27 cases with no features of MVI. Within the 12 cases with MVI, 10 cases were classified into M1, 2 cases were classified into M2. 8, 20, 8, and 3 cases were classified into WHO/ISUP grades 1, 2, 3, and 4 separately.

### ASL Imaging

**Figure 1:**
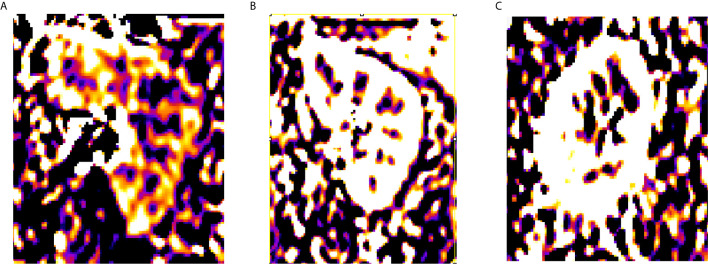
RBF of volunteers. **(A)** PLD = 500 ms, **(B)** PLD = 1,000 ms, **(C)** PLD = 2,000 ms.

Perfusion weighted images were obtained from the ASL MRI in 32 kidneys for volunteers and 39 renal lesions for 39 patients. In ASL 1, 32 kidneys were calculated for cortical RBF; the mean cortical RBF for the kidneys were 1,207.2 ± 715 ml/min/100 g (PLD1), 1,498 ± 1,053.4 ml/min/100 g (PLD2), 2,093.2 ± 1,316 ml/min/100 g (PLD3), and only 14 kidneys were successfully calculated for medulla RBF in PLD1; 12 kidneys were successfully calculated for medulla RBF in PLD2, eight kidneys were successfully calculated for medulla RBF in PLD3. In ASL 2, 32 kidneys were calculated for cortical RBF; the mean cortical RBF for the kidneys were 1,236.9 ± 687.3 ml/min/100 g (PLD1), 1,598.6 ± 978.4 ml/min/100 g (PLD2), 2,167.7 ± 1,207.6 ml/min/100 g (PLD3). Because of the poor image quality, the medulla RBF was not calculated. No significant difference of the mean values of RBF between ASL 1 and ASL 2 was shown. The cortical RBF measurements showed good agreement than the medulla and relatively lower variation (**Figure 1**). The ICC values for cortical and medulla RBF were 0.903 and 0.476 respectively.

**Figure 2:**
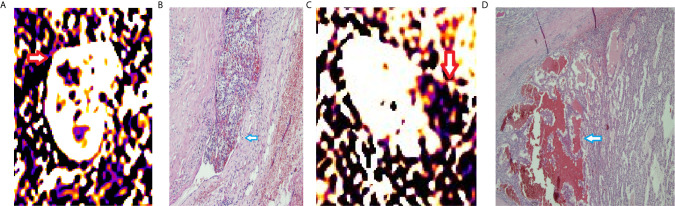
RBF of ccRCCs with or without microvascular invasion. A 50year-old man with a mass on right kidney **(A, B)**. **(A)** RBF image showed a high-perfusion mass located on the upper section of right kidney (red arrow), with central low-perfusion, indicated the central necrosis. **(B)** Pathological section showed the cancer embolus (blue arrow) in the microvascular cavity. A 63year-old woman with a mass on left kidney **(C, D)**. **(C)** RBF image showed a low-perfusion mass located on the left kidney (red arrow). **(D)** Pathological section showed no microvascular invasion. There was hemorrhage within the tumor (blue arrow).

The perfusion of the entire tumor was 867.4 ± 248.5 ml/min/100 g (PLD1), 1,085.3 ± 146.9 ml/min/100 g (PLD2), 1,704.6 ± 277.5 ml/min/100 g (PLD3). The perfusion of the solid part of the tumor was 1,251.1 ± 323.5 ml/min/100 g (PLD1), 1,618.7 ± 426.5 ml/min/100 g (PLD2), 2,101.4 ± 344 ml/min/100 g (PLD3). Mean perfusion of the solid part of the tumors with microvascular invasion was 536.4 ± 154.8 ml/min/100 g (PLD1), 2,912.5 ± 939.3 ml/min/100 g (PLD2), 3,280.3 ± 901.2 ml/min/100 g (PLD3). Mean perfusion of the solid part of the tumors without microvascular invasion was 453.5 ± 87.2 ml/min/100 g (PLD1), 1,043.6 ± 695.8 ml/min/100 g (PLD2), 1,577.6 ± 1,085.8 ml/min/100 g (PLD3) (**Figure 2**). These two groups have significant differences at all the PLDs (p < 0.05).

### Diagnostic Performance

The diagnostic performance of the entire tumor perfusion for predicting microvascular invasion of the ccRCC was assessed; the AUC was 0.778 (PLD1), 0.741 (PLD2), 0.556 (PLD3); thus the RBF of PLD1 was used to access diagnostic performance: sensitivity 75%, specificity 56%, positive predictive value 45%, and negative predictive value 88%. The diagnostic performance of the solid part of tumor perfusion for predicting MVI of the ccRCC was assessed; the AUC was 0.967 (PLD1), 0.924 (PLD2), 0.861 (PLD3); thus the RBF of PLD1 was used to access diagnostic performance: sensitivity 75%, specificity 100%, positive predictive value 66.7%, and negative predictive value 95.7%.

Among MRI characteristics, ill-defined margin and the maximum diameter of the tumor were correlated with MVI. The tumor ISUP grade has no significant difference between tumors with or without MVI. Logistic regression analysis indicated the maximum diameter of the tumor (OR = 2.934, p < 0.01), ill-defined margin (OR = 1.467, p < 0.01), the solid part of tumor perfusion (OR = 2.245, p < 0.01) were the independent prediction index for RCC accompanying MVI.

## Discussion

This is the first study that used ASL MR imaging to predict microvascular invasion of T1 staging (maximum diameter ≤7 cm) renal cell carcinoma and indicated that perfusion of RCC has great diagnostic performance for predicting microvascular invasion. In the Kidney Cancer Management Guide for RCC with diameter greater than 4 cm and less than 7 cm, there is no high-quality evidence for choosing which operation procedure (RN or PN) should be conducted. It is also mentioned in the guidelines that for patients with venous tumor thrombus, radical nephrectomy is currently recommended. MVI is the former step of venous tumor thrombus. Thus, if the MVI can be predicted before operation, this will help doctors to prefer choosing radical nephrectomy as the suitable therapy; this will push the management of RCC a step further. MVI is tightly related with the invasiveness of tumor and which could be reflected from the perfusion of tumor. ASL MRI allows the non-invasive quantitative assessment of tissue perfusion without contrast media, which is safe and promising for the prediction of MVI.

The cortex RBF showed great image quality and stability in both ASL1 and ASL2; the cortex RBF elevated with the PLD increase. The medulla RBF showed poor image quality especially with longer PLD time. No significant difference between the two sections (ASL1 and ASL2) indicated good repeatability. Among MRI characteristics, the margin (clear or ill-defined) and maximum diameter of the tumor has significant difference between tumors with or without microvascular invasion.

Zhao et al. examined 139 RCC patients and aimed to explore the relationship between clinical and pathologic features with prognostic factors and indicated that microvascular invasion was a significant predictor of prognosis (24). Bernardos et al. retrospectively analyzed 153 pT3apN0cM0 kidney cancer patients and indicated that tumor necrosis and microvascular invasion were associated with tumor recurrence (25). Kim et al. reviewed 406 patients with localized or locally advanced ccRCC who underwent curative surgery and were followed up for >2 years after surgery and indicated that tumor necrosis and MVI were independent risk factors for postoperative recurrence. Besides, microvascular invasion is the former step of venous tumor thrombus; the accurate pre-operative diagnosis can make the operation strategy be more confident.

Ma et al. (21) included 108 patients with surgically resected single intrahepatic cholangiocarcinoma to explore the association between preoperative MRI and MVI and indicated that arterial phase enhancement pattern and the enhancement ratio of arterial phase edge were associated with MVI. Vincenza Granata et al. (26) assessed major and ancillary MR parameters to explore which can be correlated with microvascular invasion of HCC and indicated that progressive pattern of contrast enhancement and satellite nodules surrounding tumors indicated presence of MVI. Wang et al. (27) analyzed 92 histopathologically confirmed HCCs with diffusion kurtosis imaging and conventional MRI features for predicting of microvascular invasion and indicated irregular surrounding enhancement as well as mean kurtosis value was associated with MVI presence of HCC. Nebbia et al. included retrospectively 99 patients who were diagnosed with HCC to investigate MRI radiomics for predicting MVI status pre-surgery and indicated that the combination of T2 sequence and portal venous sequence yields the highest AUC for MVI status prediction (28). Since previous studies indicated enhancement of tumor usually correlated with MVI, this study used ASL to non-invasively evaluate perfusion. In this study, tumor with MVI has higher RBF than that without MVI, indicating that more invasive tumor had more blood supply; this was consistent with previous study.

PCASL MRI used for measuring renal cortical perfusion is feasible and reproducible. Main ASL imaging includes continuous ASL (CASL), pseudocontinuous ASL (PCASL), and pulsed ASL (PASL) based on different labeling methods; a single shorter RF pulse or a limited number of pulses are applied in PASL than in CASL and PCASL. The signal-to-noise ratio (SNR) of the PCASL method is higher than that of the PASL method because the PCASL method has longer temporal duration of the labeled bolus and higher labeled magnetization delivered than the PASL method. Since the SNR of ASL MRI is affected by both the labeling technique and respiration movement, breath-hold strategy was used in this study to overcome respiratory mismatch (23). SE-EPI is less influenced by any B0 field homogeneity than GE-EPI and has a short acquisition time. Perfusion was found to be lower for SE-based readouts than GE-based readouts. Despite the PWI-SNR being significantly lower for SE-EPI compared with other schemes, the highest tSNR of SE-EPI readout was considered to be the most reproducible scheme for the kidney cortex assessment, with a small coefficient of variation while minimizing variability for the whole-kidney perfusion assessment (13).

In this study, cortex perfusion is feasible by using ASL MRI. Cutajar et al. (29, 30) reported that cortical RBF measurement was repeatable, which was consistent with the results of the present study. In contrast, the medullary perfusion appears poorly reproducible, which might be due to the medullary perfusion complex physiology or the lower SNR conditions since the medullary perfusion obviously decreased than the cortex. In this study, cortical RBF is higher than in other studies, and the solid part of RCC is also higher than in some studies; the tendency (the solid part of RCC is similar to kidney cortex) is similar to the other studies.

Some studies reported that spherical shape tumors are more likely to be MVI-negative. In this study, the shape of RCC does not have a significant difference between MVI-negative and MVI-positive. T1 staging RCC may usually represent spherical than irregular shape; this may cause the no significant difference between the two groups. Chou et al. prospectively evaluated the association of preoperative multiphasic CT findings of HCC with MVI and found tumor margins on CT images were associated with MVI (31). The ill-defined margin may indicate the invasiveness of tumor, which has higher angiogenesis and more likely to have venous invasion.

In previous studies, the maximum diameter of the tumor was also considered to be a risk factor of MVI; tumor size increases are usually associated with the presence of MVI. This may be meaningful in different staging than only in T1 staging.

A new WHO/ISUP grading system is recommended for use by the WHO. This is a four-grade system, mainly based on the different degrees of nucleolar prominence and the presence of sarcomatoid or rhabdoid morphology (32). Esnaola et al. (33) reported a cohort of 245 with hepatic cell cancer and indicated that tumor size and tumor grade had strong correlation with MVI. Since tumors in this study were stage T1, this may lead to a small difference of ISUP grade and lead to no significant difference between the group with or without MVI.

This study has some limitations. First, the sample size is relatively small. Studies with larger sample size could be conducted to validate the result. Second, some previous studies have used other MRI imaging reflecting perfusion such as dynamic contrast enhancement to predict MVI; the diagnostic performance of ASL and other MRI imaging techniques could be compared in future studies.

This study used PCASL MR imaging to predict microvascular invasion of T1 stage (maximum diameter ≤7cm) renal clear cell carcinoma preoperatively and indicated that the PCASL MR imaging has good reproducibility for renal cortex and good diagnostic performance for predicting MVI for ccRCC, which could help doctors in making more suitable operation strategy.

## Data Availability Statement

The raw data supporting the conclusions of this article will be made available by the authors, without undue reservation.

## Ethics Statement

The studies involving human participants were reviewed and approved by the ethics committee of West China Hospital, Sichuan University. The patients/participants provided their written informed consent to participate in this study.

## Author Contributions

Guarantor of integrity of the entire study: H-MZ and BS. Protocol/project development: H-MZ. Data collection or management: D-GW, YW, Y-GB, and YY. Data analysis: D-GW and Y-TC. Manuscript writing/editing: H-MZ. All authors contributed to the article and approved the submitted version.

## Funding

This work was supported by the China National Institutes of Health [grant number: 81801671] and Sichuan Provincial Science and Technology plan Grans [grant number: 2019YFS0445].

## Conflict of Interest

The authors declare that the research was conducted in the absence of any commercial or financial relationships that could be construed as a potential conflict of interest.

## Abbreviations

MVI, microvascular invasion; ASL, arterial spin labeling; ccRCC, clear cell renal cell carcinoma.
